# Supplementary material for: Infrared nanospectroscopy reveals the molecular interaction fingerprint of an aggregation inhibitor with single Aβ42 oligomers
Source: Nat Commun. 2021 Jan 29;12:688. doi: 10.1038/s41467-020-20782-0 (PMC7846799; doi:10.1038/s41467-020-20782-0)
Supplement: Supplementary file 1 — Supplementary Information [file 41467_2020_20782_MOESM1_ESM.pdf]

Supplementary Information for

Infrared Nanospectroscopy Reveals the Molecular Interaction Fingerprint of an Aggregation Inhibitor with Single Aβ42 Oligomers

Francesco Simone Ruggeri et al.

SUPPLEMENTARY FIGURES

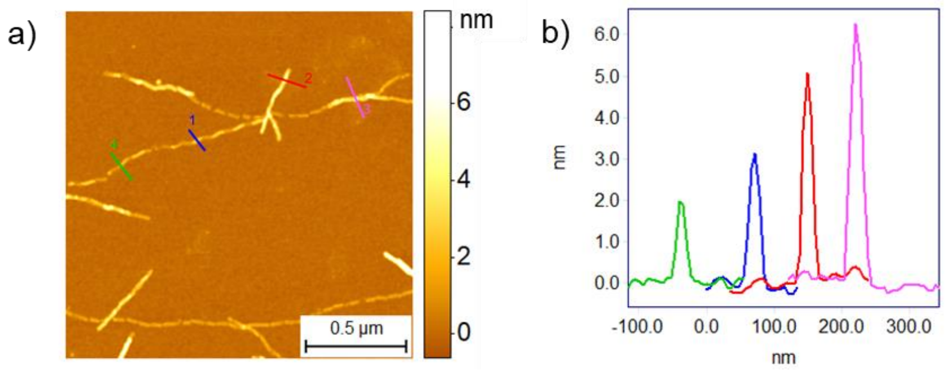

Supplementary Figure 1. Cross-sectional dimensions of Aβ42 fibrils on mica substrate. a) High resolution 3-D morphology map. b) Cross-sectional height of the aggregates. The morphology of the fibrillar aggregates was reproducible and consistent in 5 independent and randomly chosen areas on the surface.

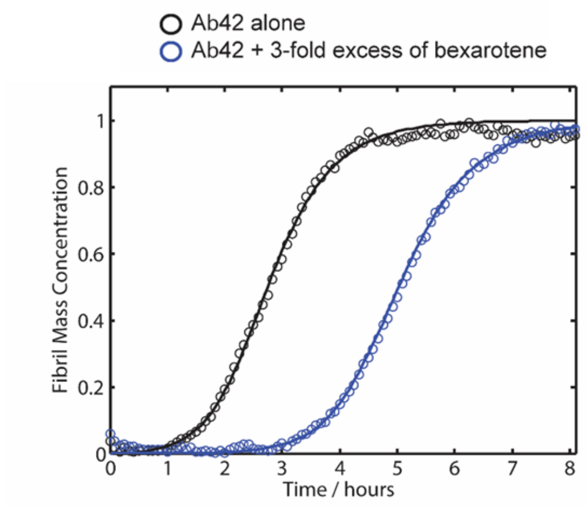

Supplementary Figure 2. Kinetics of aggregation of Aβ42 in presence of bexarotene. Kinetic profiles of the aggregation reaction of 2 µM Aβ42 in the absence or in the presence of a 3:1 concentration ratio of bexarotene to Aβ42. The solid lines show the best fit of the experimental data (open circles) when primary and secondary pathways are both inhibited by bexarotene.

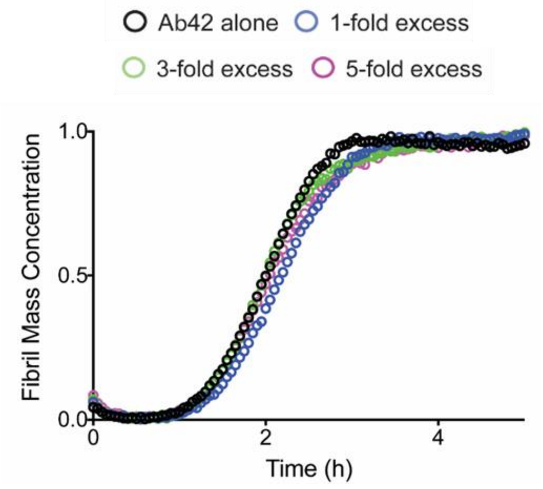

Supplementary Figure 3. Kinetics of aggregation of Aβ42 in presence of a derivative of bexarotene. Kinetic profiles of the aggregation reaction of 2 µM Aβ42 in the absence or in the presence of 1-fold to 5-fold excess of a derivative of bexarotene. Note that no significant delay in the aggregation is observed in the presence of this compound.

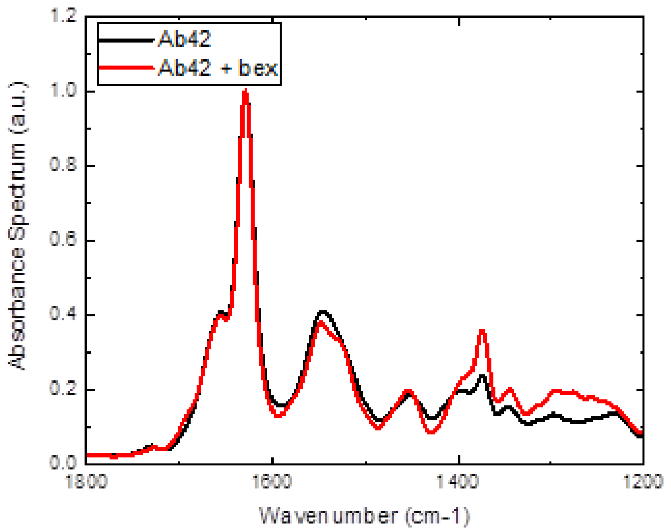

Supplementary Figure 4. ATR spectroscopy of Aβ42 fibrillar aggregates in absence and presence of bexarotene. The sample is prepared at a concentration of Aβ42 of 2 mM, which is far beyond the capabilities of conventional bulk FTIR. Thus, we acquired the spectra in air conditions, which did not allow to observe any difference between the aggregates with and without the small molecule.

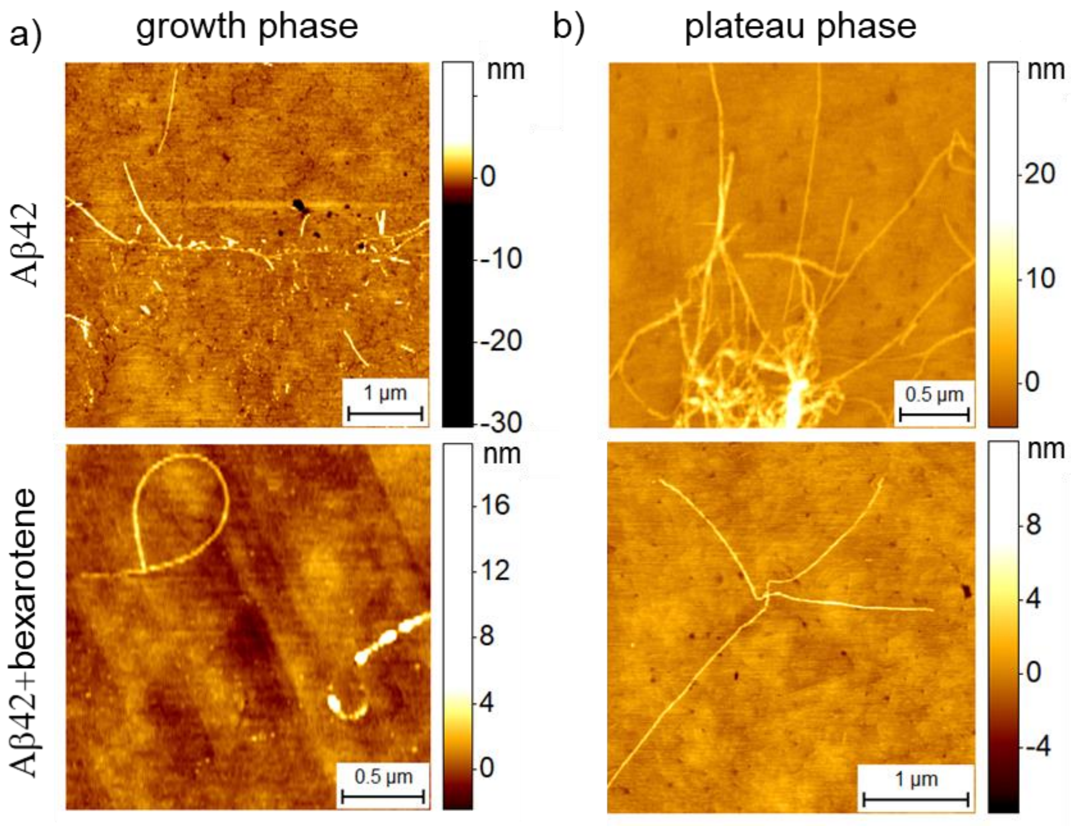

Supplementary Figure 5. Morphology of Aβ42 aggregates by AFM. Aggregates in the a) growth and b) plateau phase of aggregation on gold substrates with and without bexarotene. The morphology of the aggregates was reproducible and consistent on at least 5 independent and randomly chosen areas on the surface.

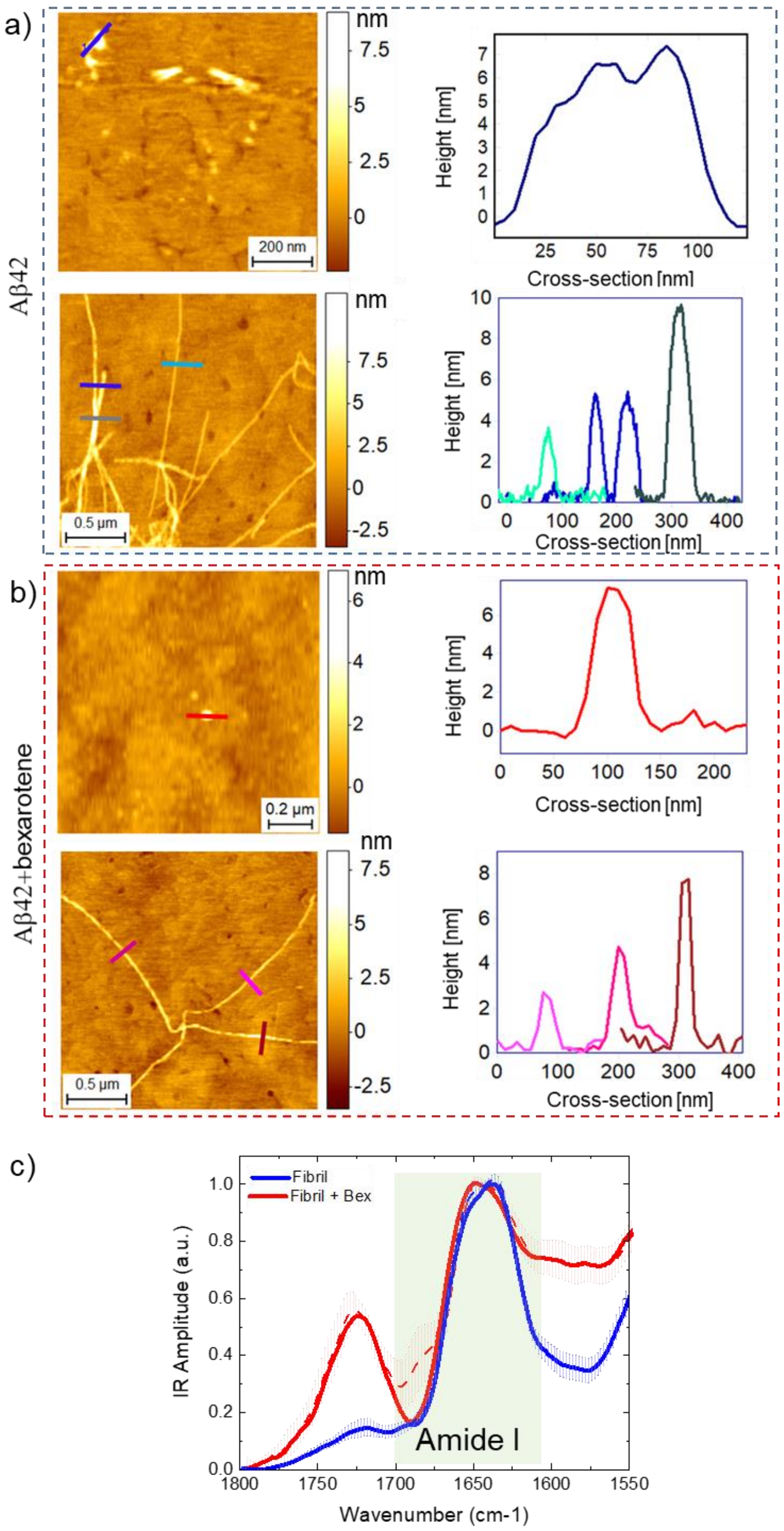

Supplementary Figure 6. Comparison of morphological and spectroscopic properties of the aggregates. AFM maps and rross-sectional dimensions of Aβ42 aggregates incubated in: a) absence and b) presence of bexarotene on gold substrates, where the AFM-IR spectra were acquired. c) Spectroscopic IR absorption of fibrillar aggregates in absence and presence of bexarotene in the amide I band. We represent two spectra: i) the average + SE of the raw spectra, and ii) the average + SE of the preprocessed spectra by applying an adjacent averaging filter (5 pts) and a Savitzky-Golay filter (2nd order, 15 pts). The number of averaged spectra for fibrils is n=24 in absence and n=20 in presence of bexarotene.

Aβ42

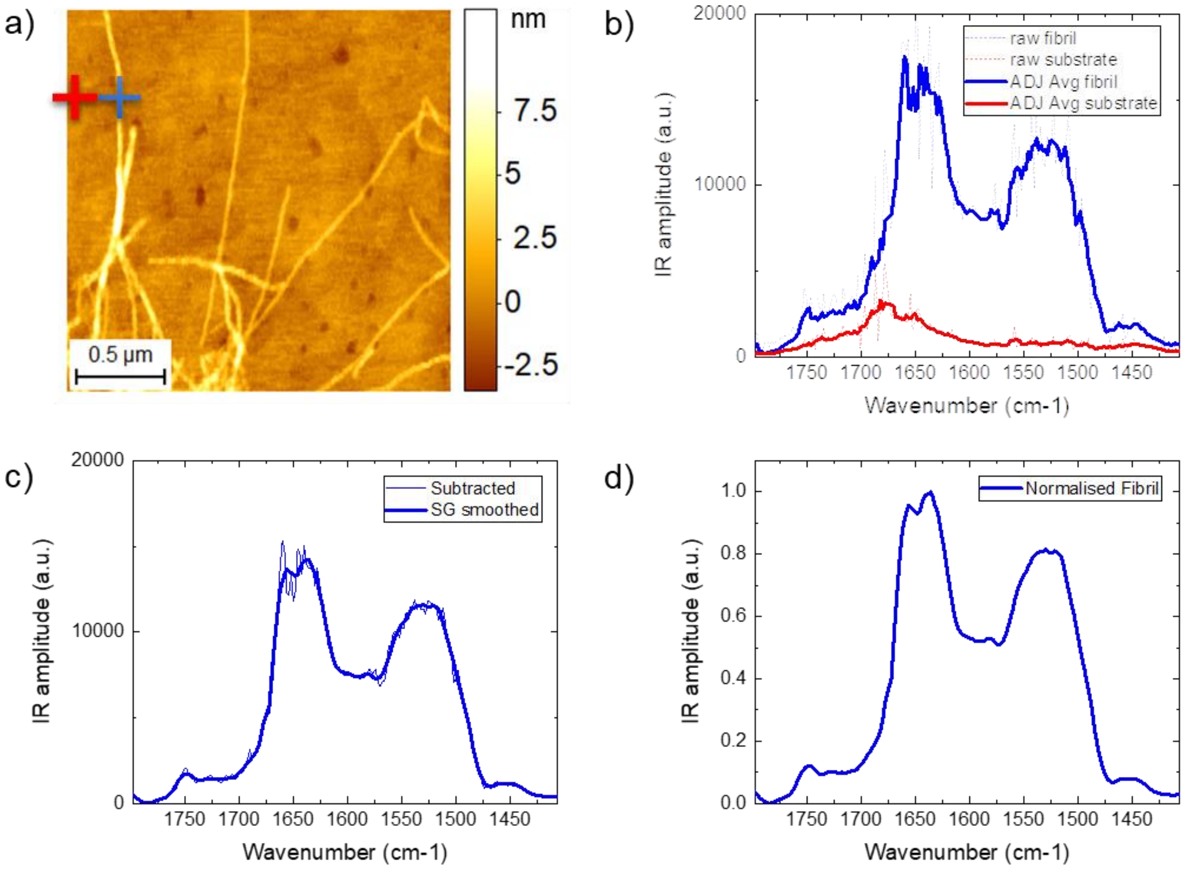

Supplementary Figure 7. Acquisition and post-processing of AFM-IR spectra. a-b) Nanoscale IR spectra are acquired on the top of the aggregate of interest (blue cross) and on the surrounding substrate (red cross). c) The two spectra are subtracted to remove tip contamination and residual substrate absorbance from the spectrum of the aggregate, which is then smoothed by a Savitzky-Golay (15 pts, 2nd order) filter, and then d) normalised to the maximum of absorption.

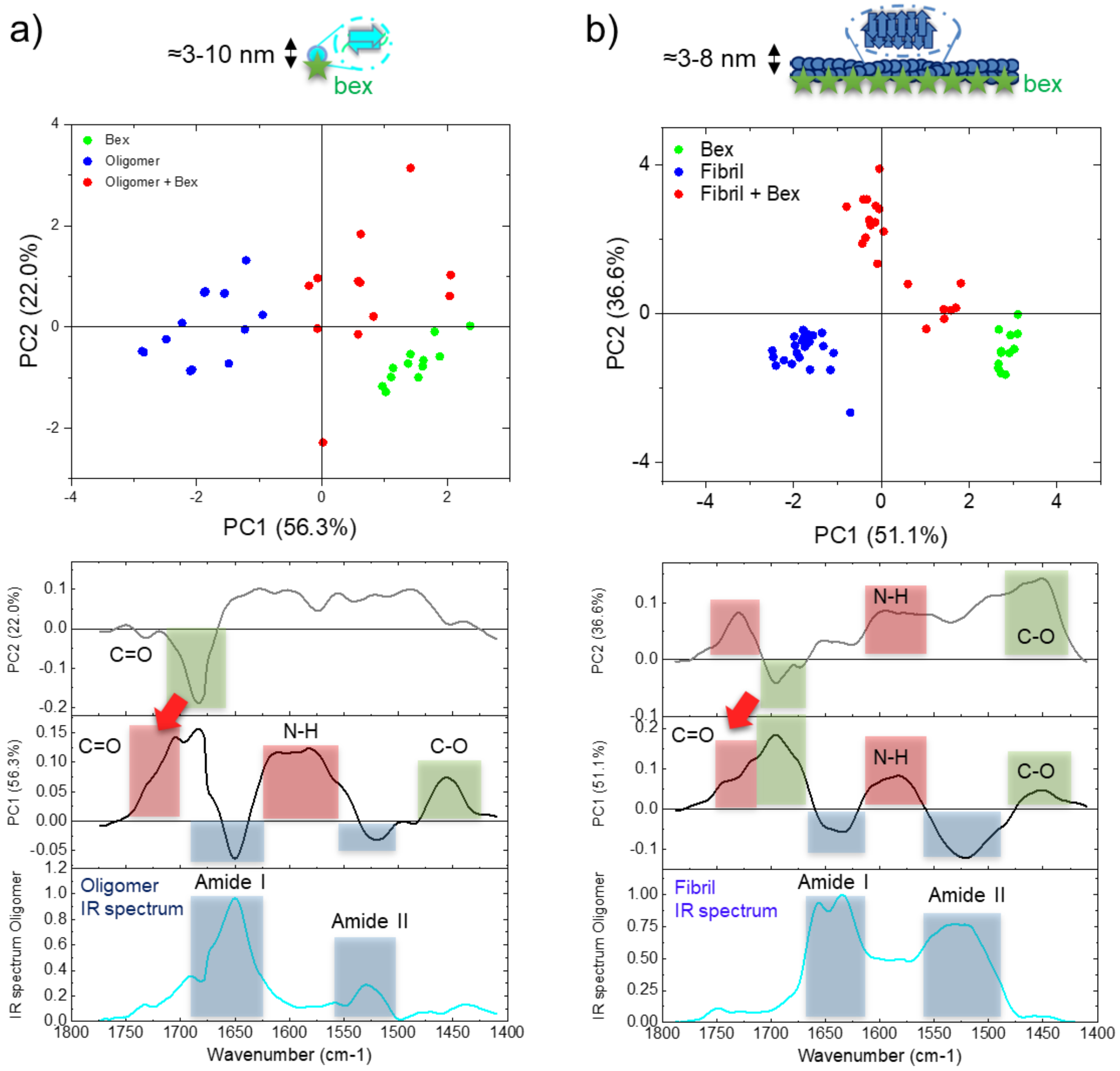

Supplementary Figure 8. PCA analysis on oligomeric and fibrillar aggregates. Score plot and relative loading plots of the analysis on: a) oligomeric species and bexarotene, b) fibrillar species and bexarotene.

a)

No. of H-bonds	Type of H-bond	H-bond dissociation energy (kJ/mol)	ν_{CO} (cm^{-1})
0	HOCO	0.0	1776.4
1	HOCO	11.1	1787.8
1	HOCO	18.3	1740.6
1	HOCO	31.3	1744.1
2	HOCO	63.7	1705.5

b)

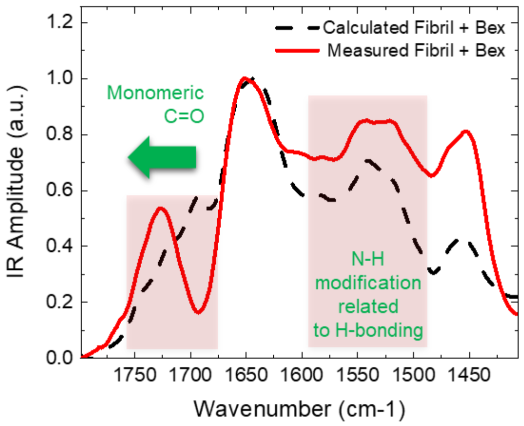

Supplementary Figure 9. Bexarotene interacts with Aβ42 aggregates through hydrogen bonding of its carboxyl group. (a) Energies and wavenumbers characteristic of different coordination states of a carboxyl group[1]. If we consider the bidentate and monomeric forms, we see that the IR band shifts from 1705.5 cm^{-1} to 1740.6-1744.1 cm^{-1} (green rows). (b) The same behaviour is found in our experimental results, where we observed a shift of the C=O stretching from ~1700 cm^{-1} to ~1730 cm^{-1} between the spectra of bexarotene alone and of the aggregates incubated with the small molecule (green arrow). b) To further validate this result, we calculated the sum of the measured spectra of bexarotene and of the amyloid aggregates alone. This calculated spectrum differs significantly from the spectrum measured on the aggregates incubated with the small molecule in the region of the carbonyl group of the molecule. The comparison of the experimental measured spectrum and of the calculated spectrum summing the experimental IR signatures of fibrils and bexarotene alone in its dimeric form confirms that the only differences in the spectra are related to a monomeric hydrogen bonding.

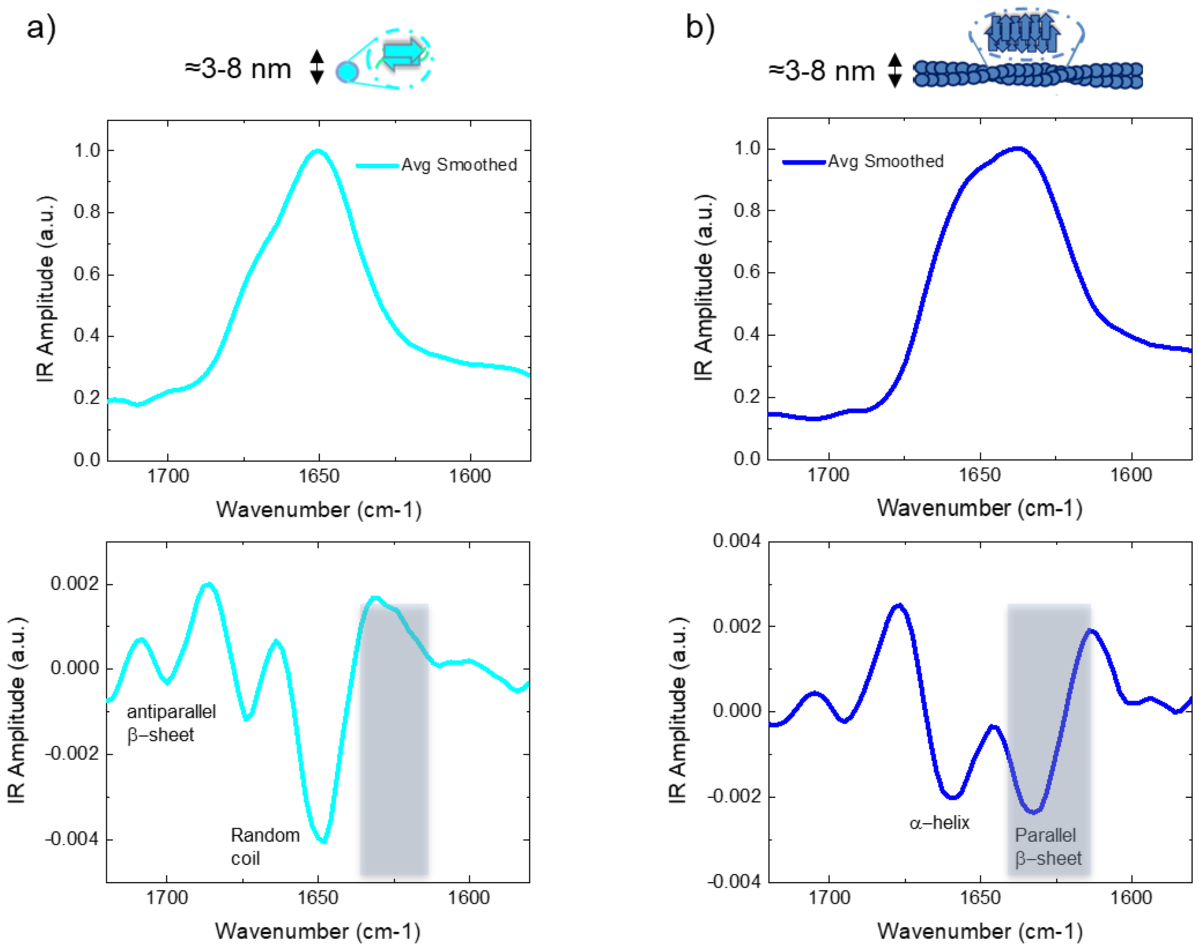

Supplementary Figure 10. IR spectra and their second derivatives for the aggregates in the Amide I region. a) Oligomers and b) fibrils.

SUPPLEMENTARY REFERENCES

1 Nie, B., Stutzman, J. & Xie, A. A Vibrational Spectral Maker for Probing the Hydrogen-Bonding Status of Protonated Asp and Glu Residues. *Biophys J* **88**, 2833-2847, (2005).
