# Supplementary material for: Infrared nanospectroscopy reveals the molecular interaction fingerprint of an aggregation inhibitor with single Aβ42 oligomers
Source: Nat Commun. 2021 Jan 29;12:688. doi: 10.1038/s41467-020-20782-0 (PMC7846799; doi:10.1038/s41467-020-20782-0)
Supplement: Supplementary file 3 — Reporting Summary [file 41467_2020_20782_MOESM3_ESM.pdf]

nature research

Corresponding author(s): Francesco Simone Ruggeri

Last updated by author(s): 2020/11/10

Reporting Summary

Nature Research wishes to improve the reproducibility of the work that we publish. This form provides structure for consistency and transparency in reporting. For further information on Nature Research policies, see our Editorial Policies and the Editorial Policy Checklist.

Statistics

For all statistical analyses, confirm that the following items are present in the figure legend, table legend, main text, or Methods section.

n/a	Confirmed	
☐	☒	The exact sample size (*n*) for each experimental group/condition, given as a discrete number and unit of measurement
☐	☒	A statement on whether measurements were taken from distinct samples or whether the same sample was measured repeatedly
☒	☐	The statistical test(s) used AND whether they are one- or two-sided<br>*Only common tests should be described solely by name; describe more complex techniques in the Methods section.*
☐	☒	A description of all covariates tested
☐	☒	A description of any assumptions or corrections, such as tests of normality and adjustment for multiple comparisons
☐	☒	A full description of the statistical parameters including central tendency (e.g. means) or other basic estimates (e.g. regression coefficient) AND variation (e.g. standard deviation) or associated estimates of uncertainty (e.g. confidence intervals)
☒	☐	For null hypothesis testing, the test statistic (e.g. *F*, *t*, *r*) with confidence intervals, effect sizes, degrees of freedom and *P* value noted<br>*Give P values as exact values whenever suitable.*
☒	☐	For Bayesian analysis, information on the choice of priors and Markov chain Monte Carlo settings
☐	☒	For hierarchical and complex designs, identification of the appropriate level for tests and full reporting of outcomes
☒	☐	Estimates of effect sizes (e.g. Cohen's *d*, Pearson's *r*), indicating how they were calculated

Our web collection on statistics for biologists contains articles on many of the points above.

Software and code

Policy information about availability of computer code

Data collection	Conventional AFM maps were collected by the commercial built-in software XEI (version 4.3.3) of the NX1O AFM (Park Systems, South Korea). Bulk FTIR spectra were collected by the built-in commercial software OPUS (version 8.2) of the FTIR Vertex 70 instrument (Bruker, USA). The AFM-IR maps and spectra were collected by the commercial built-in software "Analysis Studio" (version 3.15) of the nanolR2 (Bruker, USA) instrument. The ThT fluorescence kinetics curves were collected by the commercial built-in software of the plate reader Fluostar (version 5.4) (BMGLabtech, Germany).
Data analysis	The post-processing of the AFM maps was performed by the commercial software SPIP (version 7.3.4, Image Metrology, Denmark). The analysis of ThT, FTIR, AFM-IR data and the principal component analysis (PCA) of the AFM-IR spectra were performed by the commercial software Origin PRO 2019 (USA)

For manuscripts utilizing custom algorithms or software that are central to the research but not yet described in published literature, software must be made available to editors and reviewers. We strongly encourage code deposition in a community repository (e.g. GitHub). See the Nature Research guidelines for submitting code & software for further information.

Data

Policy information about availability of data

All manuscripts must include a data availability statement. This statement should provide the following information, where applicable:

- Accession codes, unique identifiers, or web links for publicly available datasets
- A list of figures that have associated raw data
- A description of any restrictions on data availability

All data needed to evaluate the conclusions of the paper are present in the paper and the Supplementary Information file. The source data underlying Figs 1-4 and Supplementary Figs 2, 3, 4, 6 are provided as raw data in the source data file. Other data are available from the corresponding author upon request.

Field-specific reporting

Please select the one below that is the best fit for your research. If you are not sure, read the appropriate sections before making your selection.

☒ Life sciences ☐ Behavioural & social sciences ☐ Ecological, evolutionary & environmental sciences

For a reference copy of the document with all sections, see nature.com/documents/nr-reporting-summary-flat.pdf

Life sciences study design

All studies must disclose on these points even when the disclosure is negative.

Sample size

The sample size was not predetermined and it was chosen as the number of observations necessary to determine with statistical significance the difference between aggregates incubated with/without small molecule. On each protein aggregate with/without small molecule at least 3 AFM-IR spectra were co-averaged, in order to have a standard error of the average in the order of 5%. For principal component analysis, a total of 71 spectra was sufficient to detect the subgrouping of the spectra of the aggregates incubated with/without the small molecule bexarotene, out of the 90% confidence of the variance of each group.

Data exclusions

Data were not excluded from the analysis

Replication

The kinetics experiments in presence/absence of small molecule were reproduced in triplicates, all replicates were successful and consistent. The FTIR measurements were performed on three independent samples, all replicates were successful and consistent. AFM-IR measurements were performed on 5 independent samples, for each samples several independent locations on the aggregates were measured (oligomers n=8 and fibrils n=20).

Randomization

The chemical maps and spectra were acquired on randomly chosen aggregates on each sample's surface.

Blinding

Blinding was not relevant for this study because we wanted to first prove that is possible to determine the effect and binding of the drug on the aggregates by AFM-IR. The investigator knew if the protein was incubated with/without the presence of the drug to evaluate its effect on the kinetics of aggregation. Then, the group allocation of the spectroscopic signature of each aggregates during analysis was determined by Principle Component Analysis sub-grouping.

Reporting for specific materials, systems and methods

We require information from authors about some types of materials, experimental systems and methods used in many studies. Here, indicate whether each material, system or method listed is relevant to your study. If you are not sure if a list item applies to your research, read the appropriate section before selecting a response.

Materials & experimental systems

n/a	Involved in the study
☒	☐ Antibodies
☒	☐ Eukaryotic cell lines
☒	☐ Palaeontology and archaeology
☒	☐ Animals and other organisms
☒	☐ Human research participants
☒	☐ Clinical data
☒	☐ Dual use research of concern

Methods

n/a	Involved in the study
☒	☐ ChIP-seq
☒	☐ Flow cytometry
☒	☐ MRI-based neuroimaging
